# Allicin prevents oxidized low-density lipoprotein-induced endothelial cell injury by inhibiting apoptosis and oxidative stress pathway

**DOI:** 10.1186/s12906-016-1126-9

**Published:** 2016-05-20

**Authors:** Xiaoshu Chen, Sunian Pang, Jianfeng Lin, Jianlan Xia, Yi Wang

**Affiliations:** Department of Cardiology, Wenzhou People’s Hospital, No. 57, Canghou Street, Wenzhou, 325000 China

**Keywords:** Allicin, Oxidized low-density lipoprotein, Endothelial cell, Apoptosis, Oxidative stress

## Abstract

**Background:**

Vascular endothelial apoptosis is significantly associated with atherosclerosis and cardiovascular diseases, for which oxidized low-density lipoprotein (ox-LDL) is a major risk factor. Allicin, the primary active ingredient of garlic, has been found to have cardiovascular protective effect by changing the fatty-acid composition, but its effect on ox-LDL-induced vascular endothelial injury remains unclear. We investigated the protective effect of allicin on cell viability, LDH release, apoptosis and apoptotic signaling in human umbilical vein endothelial cells (HUVECs).

**Methods:**

In cultured HUVEC cell line, ox-LDL induced injury was investigated. The cell viability and injury were evaluated by using cell proliferation Assay kit and LDH release assay. The apoptosis was evaluated by the Annexin V-FITC kit. The activity of caspase-3 was assessed using a colorimetric caspase-3 assay kit. The ROS production was evaluated by fluorometric assay and NADPH oxidase activity was assessed with a GENMED kit.

**Results:**

Exposure of HUVECs to ox-LDL (150 μg/ml) reduced cell viability, induced apoptosis and increased activity of caspase-3, NADPH oxidase, and reactive oxygen species (ROS) production. The pretreatment with allicin (30 and 100 μM) significantly rescued the cell viability, inhibited ox-LDL-induced apoptosis and activity of caspase-3, NADPH oxidase and ROS production in HUVECs, and the protective effect is concentration-dependent. The allicin (100 μM) alone did not show significant difference from control. Our study demonstrated that allicin protected HUVECs from ox-LDL-induced endothelial injury by reducing the apoptosis, mediated by inhibition of caspase-3 and NADPH oxidase related apoptotic signaling.

**Conclusions:**

Allicin prevents ox-LDL-induced endothelial cell injury by inhibiting apoptosis and oxidative stress pathway.

## Background

Cardiovascular diseases (CVDs) are medical conditions often associated with severe outcomes, financial burdens, and high mortality rate worldwide [1, 2]. It is found that atherosclerosis significantly involved in the pathogenesis of CVDs, during which lipids and plaques accumulate in large arteries [3] and induce endothelial dysfunction during the development of atherosclerosis [4]. Endothelial injuries can be contributed by various factors such as oxidized low-density lipoprotein (ox-LDL), advanced glycation end-productcs (AGEs), and Angiotensin-2 etc. [5]. Ox-LDL disrupts the antioxidant and secretory activities of vascular endothelium and induces endothelial apoptosis, thus being considered an important risk factor for endothelia injury [6]. Meanwhile, the increased superoxide in vessels during oxidative stress facilitates the oxidation of LDL and generates higher amount of ox-LDL, which cause further detrimental effect on endothelial functions [7].

Natural antioxidants from food sources can be wholesome remedies in preventing and treating atherosclerosis with relatively smaller side effects, among which garlic has garnered significant attention because of its cardiovascular benefits [8]. Allicin (2-propene-1-sulfinothioic acid S-2-propenyl ester), the primary active ingredient in garlic, has been found to produce cardiovascular protection by changing the fatty acid composition in high-fat-diet mice and rats [9], while its direct action on ox-LDL-induced vascular endothelial damage remains unknown.

Therefore, we constructed an in vitro oxidative stress model subjected to ox-LDL-exposure and investigated the effect of allicin on ox-LDL-induced vascular endothelial injury. Since ox-LDL-induced endothelial cell apoptosis is significantly involved in the progress of atherosclerosis [10], and because reactive oxygen species (ROS) play crucial roles in the process of cell apoptosis [10], we explored the underlying mechanism for the protective effect of allicin by studying whether it reduces ox-LDL-mediated ROS production and regulates the ox-LDL triggered apoptotic pathway such as caspase-3 expression in endothelia cells.

## Methods

### Materials

HUVEC cell line was originally acquired from the American Type Culture Collection (Manassas VA, USA). Native LDL (nLDL) was purchased from Sigma-Aldrich (St. Louis, Mo., USA). Dulbecco’s modified Eagle’s media (DMEM), dimethylsulfoxide (DMSO), and allicin were obtained from Sigma (St. Louis, MO, USA). LDH assay kit was purchased from Nanjing Jiancheng Bioengineering Institute (Nanjing, China). The AnnexinV-FITC kit was purchased from Invitrogen (Carlsbad, CA, USA). All other reagents were of analytical grade and purchased from Sigma-Aldrich (St. Louis, MO, USA).

### Preparation of oxLDL [11]

nLDL was incubated with CuSO_4_ (10 μM) at 37 °C for 24 h. The material was dialyzed against a sterile solution with NaCl (150 mM), EDTA (1 mM), polymixin B (100 μg/ml), and pH at 7.4. The presence of oxLDL was confirmed by agarose gel electrophoresis and the generation of thiobarbituric acid.

### Cell culture and treatment

Endothelial cell line HUVECs were cultured in DMEM (low glucose) supplemented with 10 % fetal bovine serum, and maintained in a humidified atmosphere containing 5 % CO_2_ at 37 °C. The cells between passages 2 and 15 were used in this study.

For the treatment, HUVECs were pre-treated for 20 min with allicin at different concentrations (10, 30, 100 μM) and then exposed to ox-LDL (150 μg/ml) for the indicated time periods. Since allicin was dissolved in DMSO, we used 0.1 % DMSO as vehicle control. In our pilot study, we found that this concentration of DMSO had no effect on the cells in the presence or absence of ox-LDL.

### Cell viability and LDH release assay [12]

The cell viability was evaluated by using the Cell Titer 96® AQueous One Solution Cell proliferation Assay kit (Promega Corporation, Madison, WI, USA), which includes a novel aqueous soluble tetrazolium compound, MTS, and an electron coupling reagent, phenazine ethosulfate. The MTS turns to formazan product by mitochondrial dehydrogenase enzymes in active cells. The formazan product is soluble in the medium and measured spectrophotometrically at 490 nm. The absorbance is proportional to the number of viable cells in the cell culture.

During the treatment, HUVECs were cultured in 96-well plates at a density of 1 × 10^4^ per well and were allowed to grow to the desired confluence. The cells were then pre-treated for 20 min with allicin (10 – 100 μM) and then exposed to ox-LDL (150 μg/ml) for 24 h. Cell viability was normalized to the control group. The LDH released into the media were assessed to determine LDH activity using an analysis kit according to the manufacturer’s instructions.

### Flow cytometry

The percentage of apoptotic cells was evaluated by flow cytometry using the Annexin V-FITC kit following manufacturer’s instructions [13]. Briefly, HUVECs were cultured in 6-well plates at a density of 2 × 10^5^ cells per well and treated with allicin (10, 30, 100 μM) for 20 min prior to 24 h of ox-LDL exposure. Attached cells were harvested by trypsinization and incubated with annexinV-FITC and propidium iodide (PI) for 15 min at room temperature in the dark. The cell apoptotic rates were then quantified by flow cytometry.

### Measurement of caspase-3 activity

The activity of caspase-3 like protease in the lysate was assessed using a colorimetric caspase-3 assay kit (Sigma, St. Louis, MO, USA) according to the manufacturer’s protocol [14]. In brief, the reaction mixture (total volume, 100 μl) that contained 30 μl of cell lysate and 10 μl of caspase-3 substrate acetyl–Asp–Glu–Val–Asp–p-nitroanilide (final concentration at 200 μM) in assay buffer was used, and the assay was performed in 96-well plate. The mixture was incubated for 90 min at 37 °C and the absorbance was measured at 405 nm. The caspase-3 activity was expressed by value of OD405 relative to control, which was set to 100.

### Measurement of ROS production

The effect of allicin on ROS production in HUVECs was evaluated by fluorometric assay using DHE as a probe for the presence of superoxide [14]. After preincubation for 2 h with allicin at the indicated concentrations, HUVECs were incubated with DHE for 1 h, followed by incubation with ox-LDL for 2 h. The fluorescence intensity was measured at 540-nm excitation and 590-nm emission (before and after exposure to oxLDL) using a fluorescence microplate reader (Labsystems). The percentage increase in fluorescence per well was calculated by the formula [(Ft_2_ − Ft_0_)/Ft_0_] × 100, where Ft_2_ is the fluorescence at 2 h of ox-LDL exposure, and Ft_0_ is the fluorescence at 0 min of oxLDL exposure.

### Quantitative determination of NADPH oxidase activity by colorimetric analysis

NADPH oxidase activity was assessed with a GENMED kit (Genmed Scientifics Inc., Shanghai, China) by colorimetric method [15]. HUVECs were plated (5 × 10^6^/mL) in six-well plates with EGM-2 medium containing 2 % FBS. After 24 h, ox-LDL was added and cells were cultured for additional 24 h. Thereafter, the protein was extracted from cells and NADPH oxidase activity was evaluated by GENMED kit according to the manufacturer’s instruction.

### Statistical analysis

All data are presented as mean ± S.D of results from three independent experiments. The significance of the difference was analyzed by ANOVA followed by Newman-Student-Keuls test. A value of *P* < 0.05 was considered statistically significant.

## Results

### Allicin increased cell viability and inhibited LDH release in ox-LDL-exposed HUVECs

**Fig. 1:**
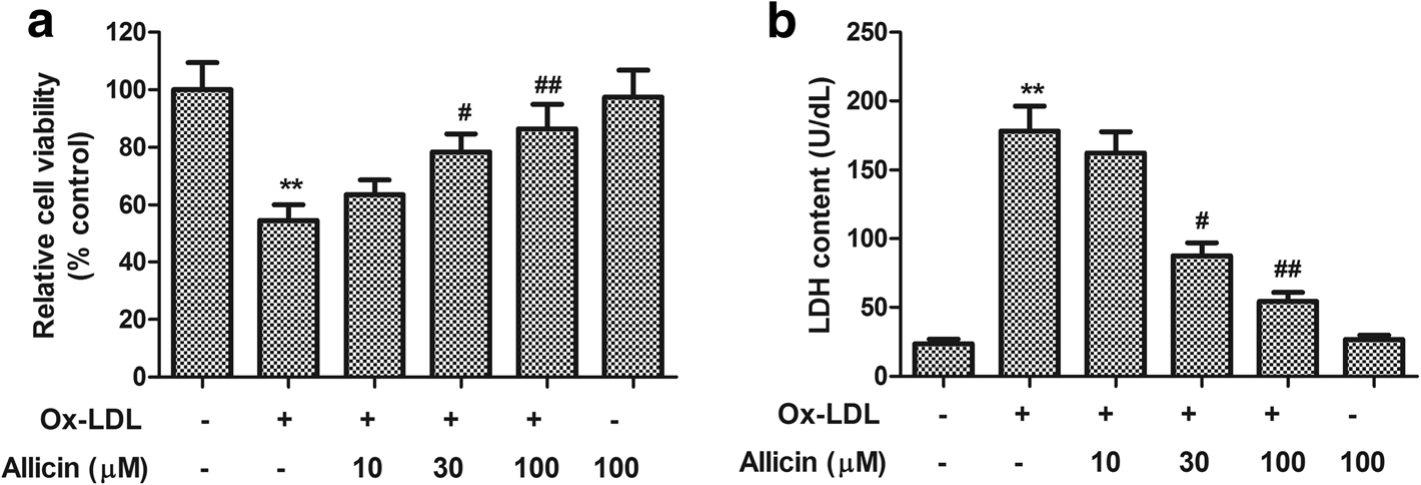
Effects of allicin on ox-LDL-induced reduced cell viability and increased LDH release in HUVECs. HUVECs were pre-treated for 20 min with allicin at different concentrations (10, 30, 100 μM), and then exposed to ox-LDL (150 μg/ml) for 24 h. Cell viability was evaluated by the MTS method and LDH content in media was measured by assay kit. **a** Relative cell viability measured by MTS and **b** LDH content in media. Values are means ± SD from three independent experiments. ^**^ *P* <0.01 vs. control, ^#^ *P* <0.05, ^##^ *P* <0.01 vs. ox-LDL

HUVEC cell viability and LDH content in culture medium were quantified to assess whether allicin protects endothelial cells from ox-LDL-induced injuries. As shown in Fig. 1, 24 h of exposure to ox-LDL (150 μg/ml) significantly decreased the HUVEC cell viability and promoted LDH release, compared with normal control (cell viability: control 100.0 % vs. ox-LDL 54.6 ± 5.4 %; LDH content: control 23.6 ± 3.3 U/dL vs. ox-LDL 178.5 ± 17.6 U/dL, *P* < 0.01). The pretreatment with allicin significantly rescued the cell viability and suppressed the LDH release in a concentration-dependent manner, which demonstrates the anti-cytotoxic activity of Allicin. On the other hand, allicin alone (100 μM) had no effect on cell viability and LDH release in HUVECs.

### Allicin inhibited ox-LDL-induced apoptosis in HUVECs

**Fig. 2:**
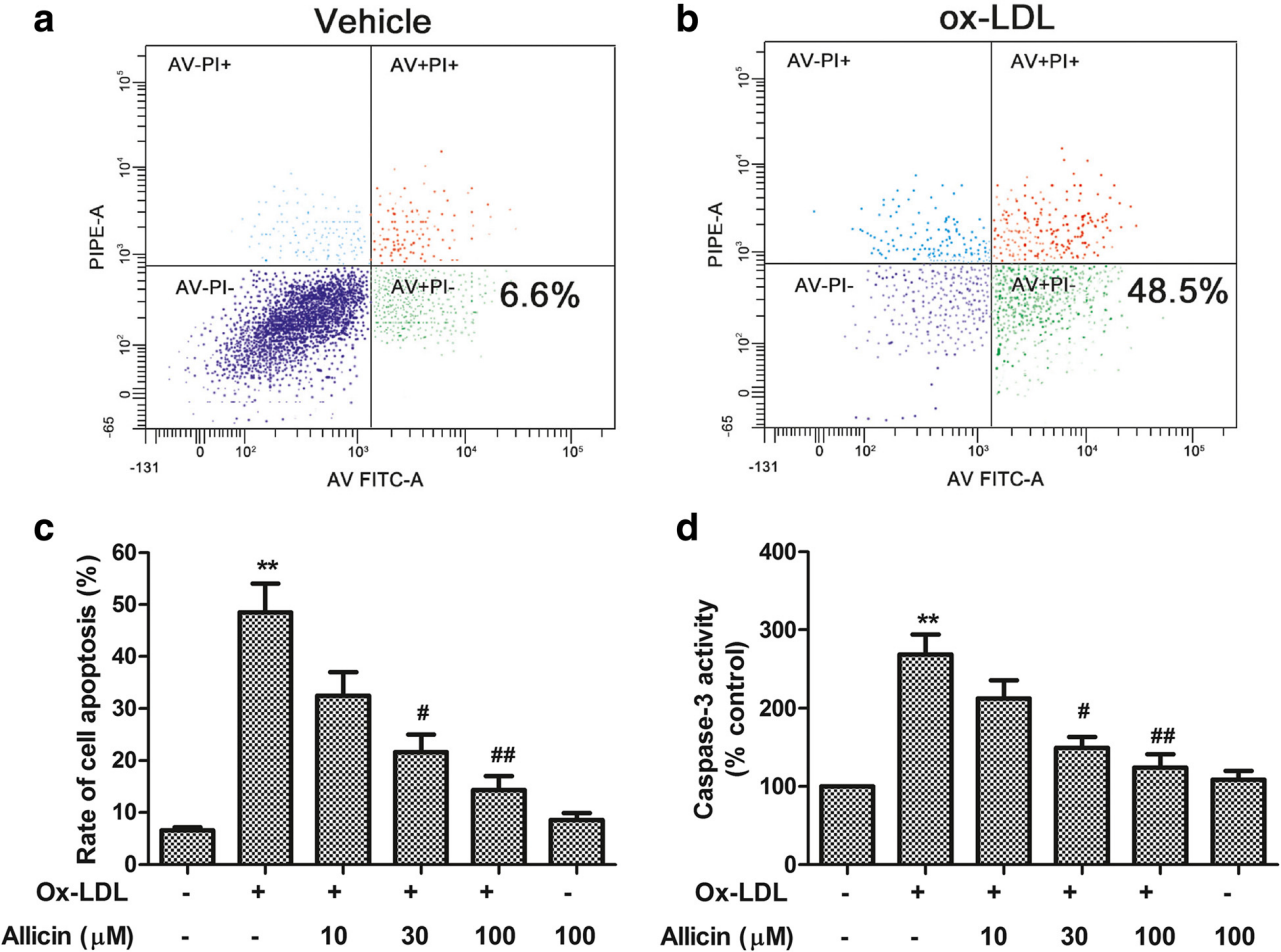
Inhibitory effects of allicin on ox-LDL-induced HUVECs cell apoptosis and caspase-3 activation. Cells were pre-treated for 20 min with allicin at different concentrations (10, 30, 100 μM), and were exposed to ox-LDL (150 μg/ml) for 24 h. Cell apoptosis was measured by Flow cytometry. **a**, **b** Flow cytometry dot plot figures of apoptotic cells in control (**a**) and ox-LDL-exposed group (**b**). **c** Rate of apoptotic cells quantified by flow cytometry. **d** Caspase-3 activity relative to control that was set as 100. Values are means ± SD from three independent experiments. ^**^ *P* <0.01 vs. control, ^#^ *P* <0.05, ^##^ *P* <0.01 vs. ox-LDL

To investigate the anti-apoptotic effects of allicin on ox-LDL-exposed HUVECs, annexin V/PI double staining and flow cytometry analysis were performed. As seen in Fig. 2a and b (representative images for flow cytometry and the summarized data), ox-LDL significantly increased the HUVEC apoptosis rate from 6.6 % to 48.5 % compared with control (*P* < 0.01). Pre-treatment with allicin (10, 30, 100 μM) significantly reduced the apoptosis in a concentration-dependent manner (Fig. 2c). Similar to the neutral impact on cell viability and LDL release, allicin (100 μM) alone had no effect on the apoptosis (Fig. 2c).

Subsequently, our study zoomed in on the effect of allicin on expression of caspase-3, the typical caspase that plays a central role in cell apoptosis. We found that normal control endothelial cells had relatively minimal expression of cleaved caspase-3. However, 24 h of ox-LDL-exposure markedly increased the expression and activity of caspase-3 (*P* < 0.01) (Fig. 2d). Pre-treatment of endothelial cells with allicin (10–100 μM) inhibited the ox-LDL-induced caspase-3 activation (Fig. 2d), during which allicin at higher concentration demonstrated stronger effect than that of lower concentration, while allicin (100 μM) alone again had no effect on cleaved caspase-3 activity.

### Allicin suppressed the ROS generation and NADPH oxidase activation in ox-LDL-exposed HUVECs

**Fig. 3:**
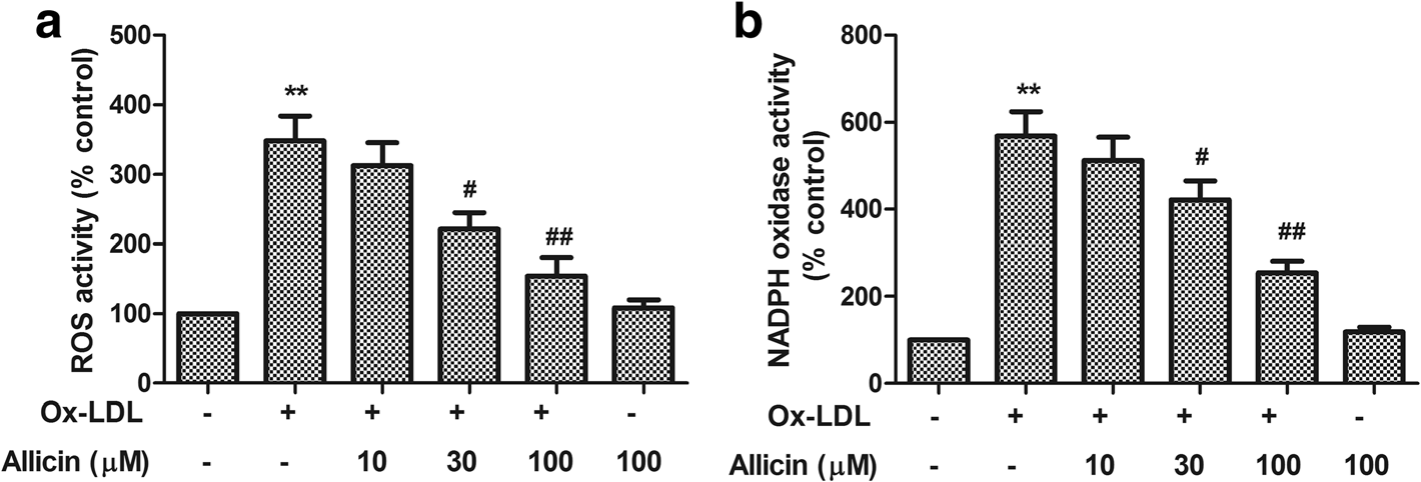
Inhibitory effect of allicin on ox-LDL-induced ROS overproduction and NADPH oxidase activation. **a** After pre-treated with allicin (10, 30, 100 μM) for 20 min, cells were exposed to ox-LDL (150 μg/mL) for 2 h followed by a 1-h incubation with superoxide-sensitive fluorescent probe DHE (10 μM). Fluorescence intensity of cells was measured with a fluorescence microplate reader. Fluorescence distribution of DHE oxidation is expressed as a percentage of increased intensity. **b** Allicin attenuated the level of NADPH oxidase activation. HUVECs were pretreated with allicin at indicated concentrations for 2 mins followed by 24 h of ox-LDL (150 μg/mL) exposure. NADPH oxidase activity was determined by colorimetric analysis. Values are means ± SD from three independent experiments. ^**^ *P* <0.01 vs. control, ^#^ *P* <0.05, ^##^ *P* <0.01 vs. ox-LDL

Considering the important role of ROS in cell apoptosis, we examined whether allicin regulates ROS generation in ox-LDL-exposed HUVECs. By using DHE as a fluorescence probe, we assessed the ROS generation and found that treatment with oxLDL (150 μg/ml) for 2 h increased ROS generation by 3.5-fold, compared with control cells, whereas the level of ROS in control group was similar to that in the 100 100 μM allicin treatment group (Fig. 3a). In addition, the generation of ROS induced by ox-LDL exposure was inhibited by allicin in a concentration dependent manner (Fig. 3a).

Since the NOX family of NADPH oxidases represents one of major source of endothelial ROS production, we evaluated the effects of allicin on NADPH oxidase activation in HUVECs after the ox-LDL exposure. We found that treatment with ox-LDL (150 μg/ml) for 24 h resulted in a 5.7-fold increase in NADPH oxidase activation compared with controls, whereas the level of ROS in control cells was similar to that in 100 μM allicin treatment group (Fig. 3b). In addition, our result demonstrated that the NADPH oxidase activation induced by ox-LDL exposure was inhibited by allicin in a concentration dependent manner (Fig. 3b).

## Discussion

The endothelium dysfunction is a critical early event in the pathogenesis of atherosclerosis [16]. It is also known that endothelial apoptosis can destabilize atherosclerotic plaques and lead to thrombosis [17], which precipitates atherosclerosis, causes acute cardiovascular symptoms, and complicates CVDs such as coronary artery disease [18].

Considering the critical role of ox-LDL in the progression of atherosclerosis and the significance of endothelial dysfunction as an early marker during this development [19], we used ox-LDL-exposed HUVECs as the model to investigate the endothelial protective effect provided by allicin.

Ox-LDL disrupts endothelial function such as endothelium secretory activity, antioxidant capabilities and nitric oxide synthesis, and induces endothelial apoptosis [20]. Thus, our exploration of the potential mechanism underlying the endothelial protective effect of allicin against ox-LDL-induced injury has been focused on its antioxidant activities, and impact on endothelial apoptosis and the major player involved in the apoptosis pathway.

First of all, our study demonstrated that allicin markedly increased cell viability in ox-LDL-exposed HUVECs and protected endothelial cells against ox-LDL-induced apoptosis in concentration-dependent manner. There are two major apoptosis pathways, one involving death receptors and the other being intrinsic or mitochondrial pathway [20]. Caspase-3 activation plays central role for both apoptotic pathways which converge at proteolytic activation of caspase-3 [13]. Our study showed that ox-LDL increased the activity of caspase-3, while we also observed that allicin decreased ox-LDL-induced caspase-3 activation, which are consistent with the results that allicin markedly prevented endothelial cells from ox-LDL-induced cell apoptosis. Thus, it is suggests that the anti-apoptotic effect of allicin on vascular endothelial cells may be mediated by the inhibition of caspase-3 activation.

The ROS production plays pivotal roles in mediating endothelial cell apoptosis and regulating the development of atherosclerosis [21], while NADPH oxidase is a major source of vascular ROS production, as the enzyme complex of NADPH oxidases are considered a major source of superoxide anion formation [22]. In addition, NADPH oxidase is also heavily involved in endothelial apoptosis [23]. In present study, we investigated the effect of allicin on ROS and NADPH oxidase activation in ox-LDL-exposed endothelial cells. Our results indicate that allicin suppressed endothelial ROS production and inhibited the NADPH oxidase activation, which helps to elucidate the underlying mechanism for the endothelial protective effect of allicin against ox-LDL-induced injury.

Our study demonstrated that the protective effect of allicin is concentration-dependent, with higher concentration producing stronger benefits against ox-LDL-induced damage, but the neutral impact from allicin (100 μM) alone on either endothelial cell viability or ROS production suggests that the anti-oxidant and endothelial protective effects of allicin are responsive to ox-LDL-induced injury, and the endothelial protection by allicin is mediated by regulation and inhibition of ox-LDL-triggered apoptotic signaling, which helped to elucidate the underlying mechanism of action of allicin and reinforced its significance as a new therapeutic agent for atherosclerosis and CVDs for which ox-LDL is a major risk factor.

There are some limitations in this work. Fristly, only ox-LDL HUVECs model was used. Whether allicin protects vascular endothelial cells from other risk factors of vascular diseases remains to be investigated further. Secondly, we only investigate inhibition of allicin on apoptosis and oxidative stress. Further mechanism should be further investigated. Thirdly, we only invested protection of allicin in vitro model. When resources allows, we will expand the study and include the investigation on in vivo models.

## Conclusions

Allicin protects vascular endothelial cells against ox-LDL-induced cell death by inhibiting the apoptotic pathway and anti-oxidative stress.

### Ethics approval and consent to participate

Not applicable.

### Consent for publication

Not applicable.

### Availability of data and materials

Not applicable.

## Abbreviations

CVD: cardiovascular disease
DHE: dihydroethidium
DMSO: dimethyl sulfoxide
HUVEC: human umbilical vein endothelial cell
LDH: lactate Dehydrogenase
NADPH: nicotinamide adenine dinucleotide phosphate
nLDL: native low-density lipoprotein
ox-LDL: oxidized low density lipoprotein
ROS: reactive oxygen species
